# Prognostic significance and function of mammalian target of rapamycin in tongue squamous cell carcinoma

**DOI:** 10.1038/s41598-017-08345-8

**Published:** 2017-08-15

**Authors:** Shau-Hsuan Li, Chih-Yen Chien, Wan-Ting Huang, Sheng-Dean Luo, Yan-Ye Su, Wan-Yu Tien, Ya-Chun Lan, Chang-Han Chen

**Affiliations:** 1 Department of Hematology-Oncology, Kaohsiung Chang Gung Memorial Hospital and Chang Gung University College of Medicine, Kaohsiung, Taiwan; 2 Department of Otolaryngology, Kaohsiung Chang Gung Memorial Hospital and Chang Gung University College of Medicine, Kaohsiung, Taiwan; 3 Department of Pathology, Kaohsiung Chang Gung Memorial Hospital and Chang Gung University College of Medicine, Kaohsiung, Taiwan; 4 Institute for Translational Research in Biomedicine, Kaohsiung Chang Gung Memorial Hospital, Kaohsiung, Taiwan; 5 Department of Applied Chemistry, and Graduate Institute of Biomedicine and Biomedical Technology, National Chi Nan University, Chi Nan, Taiwan; 6 Center for Infectious Disease and Cancer Research, Kaohsiung Medical University, Kaohsiung, Taiwan

## Abstract

Despite improvement in preoperative imaging, surgical technique, and adjuvant therapy, the prognosis of patients with tongue squamous cell carcinoma (SCC) is still unsatisfactory. The mammalian target of rapamycin (mTOR) play a key role in the regulation of tumor cell proliferation and survival. However, the significance of mTOR on the prognosis of tongue SCC remains largely undefined. In the present study, immunohistochemistry was performed to evaluate the expression of phosphorylated mTOR (p-mTOR) in 160 surgically resected tongue SCC, and correlated with survival. Univariate analysis revealed that p-mTOR overexpression (P = 0.006) was associated with inferior overall survival. In multivariate comparison, p-mTOR overexpression (P = 0.002, hazard ratio = 2.082) remained independently associated with worse overall survival. *In vitro* study, tongue cancer cells treated with everolimus, the specific mTOR inhibitor, or transfected with mTOR-mediated siRNAs dramatically attenuated the abilities of cell proliferation by MTT and BrdU assays. In 4-NQO-induced tongue cancer murine model, mTOR inhibitors significantly decreased the incidence of tongue SCC. In conclusion, p-mTOR overexpression was independently associated with poor prognosis of patients with tongue SCC. *In vitro* and *vivo*, mTOR inhibition showed the promising activity in tongue SCC. Our results suggest that inhibition of mTOR signaling pathway may be a novel therapeutic target for tongue SCC.

## Introduction

Head and neck cancer is one of the ten most frequent cancers worldwide, with an estimated over 500 000 new cases being diagnosed annually^1^. More than 90% of head and neck cancers are squamous cell carcinoma (SCC)^1, 2^. In Taiwan, the incidence of head and neck cancer increased rapidly, and was ranked as the fourth leading cancer death in males in 2011^3^. Tongue cancer is a common form of head and neck cancer and accounted for 28.11% of patients in 2011 in Taiwan^3^. Tongue has a rich supply of lymphatic drainage and neurovascular bundle which increase the incidence of cervical lymph node metastasis. The presence of cervical lymph node metastasis is a particularly reliable predictor of regional and distant failure^4^. Therefore, the prognosis of tongue SCC is worse than that in the other sites of oral cavity. The treatment for early-stage tongue SCC is usually single modality, either surgery or radiotherapy. The treatment for locally advanced tongue SCC is multimodal, with either surgery followed by adjuvant radiation or chemoradiotherapy^5^. Despite advances in preoperative imaging, surgical technique, and adjuvant therapy, the survival of patients with tongue SCC is still unsatisfactory over the past several decades^6^. Therefore, discovery of prognostic biomarkers for tongue SCC may provide an useful insight that help the development of potential novel target.

The mammalian target of rapamycin (mTOR) is a serine/threonine protein kinase that plays an important role in regulating ribosomal protein translation, cap-dependent translation, and protein synthesis^7^. In response to a variety of biochemical pathways such as hypoxia, intracellular energy change, growth factor signaling molecules, hormones, and cytokines, mTOR can be activated by phosphorylation of Ser2448 through the phosphatidylinositol 3-kinase (PI3K)/AKT signaling pathway^7^. Previous studies have reported that dysregulation of mTOR signaling pathway is associated with tumorigenesis, tumor angiogenesis, metastasis, and anticancer drug resistance^7–9^. In particular, recent development of the mTOR inhibitors, rapamycin analogs such as Everolimus (Novartis, Switzerland) and Temsirolimus (Wyeth-Ayerst, PA, USA), have generated considerable excitement in both the clinical and basic cancer research communities, as it exhibits potent activity against a wide panel of cancers^10, 11^. Therefore, further evaluation of the mTOR signaling in human cancers is important for development of anti-neoplastic therapy targeting the mTOR signaling pathway. For patients with head and neck SCC, recent studies^12–14^ showed the inconsistent findings of prognostic significance of mTOR signaling activation, indicating that the effect of mTOR signaling activation may be diverse in different head and neck site. Therefore, we conducted the present study to evaluate the significance of mTOR activation on the prognosis of tongue SCC. Besides, we also investigate the functional relevance of mTOR signaling pathway *in vitro* and *in vivo*.

## Results

### Patient characteristics

**Figure 1:**
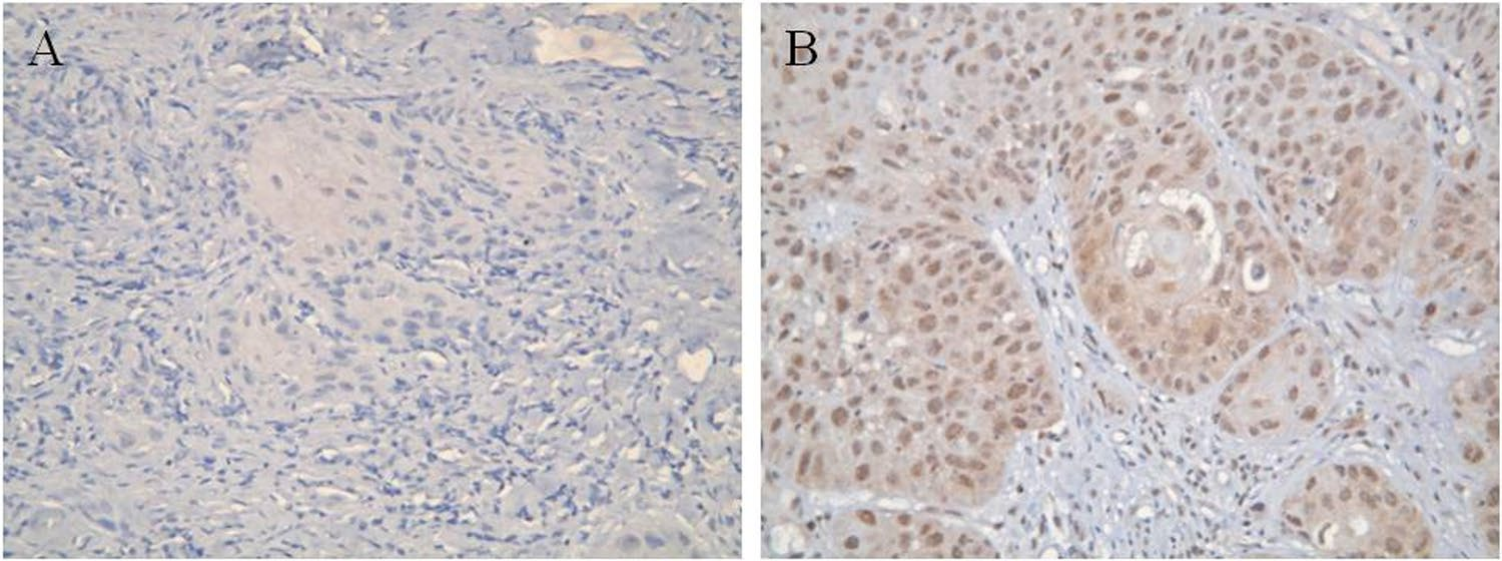
Immunohistochemical staining of p-mTOR. (**A**) Representative image of low p-mTOR expression. (**B**) Representative image of p-mTOR overexpression. Original magnification ×200.

The clinicopathologic parameters of the 160 patients with tongue SCC are described in Table 1. Among the 160 patients included in the study, p-mTOR overexpression was identified in 85 (53%) patients (Fig. 1). At the time of analysis, the median periods of follow-up were 87 months (range, 61–113 months) for the 76 survivors and 63 months (range, 1–113 months) for all 160 patients. The 5-year overall and disease-free survival rates of these 160 patients were 52% and 48%, respectively.

**Table 1:** Characteristics of 160 patients with tongue squamous cell carcinoma.

Age (years) (median: 52, range: 26–85)	
Sex	
male	147 (92%)
female	13 (8%)
Smoking	
Absent	29 (18%)
Present	131 (82%)
Alcohol	
Absent	32 (20%)
Present	128 (80%)
Betel-nut chewing	
Absent	39 (24%)
Present	121 (76%)
Pathological T classification	
T1	46 (29%)
T2	52 (32%)
T3	13 (8%)
T4a	44 (28%)
T4b	5 (3%)
Pathological N classification	
N0	87 (54%)
N1	23 (14%)
N2	47 (30%)
N3	3 (2%)
Pathological 7^th^ AJCC Stage	
I	33 (21%)
II	32 (20%)
III	24 (15%)
IVA	64 (40%)
IVB	7 (4%)
Depth of invasion (mm)	
<4 mm	20 (13%)
≥4 mm	140 (87%)
Histologic grade	
1	93 (58%)
2	62 (39%)
3	5 (3%)
p-mTOR expression	
Low expression	75 (47%)
Overexpression	85 (53%)
Vascular invasion	
Absent	132 (82%)
Present	28 (18%)
Perineural invasion	
Absent	89 (56%)
Present	71 (44%)
Extracapsular spread	
Absent	120 (75%)
Present	40 (25%)
Margin status	
Negative	148 (92%)
Positive/close	12 (8%)

Abbreviation: p-mTOR, phosphorylated mammalian target of rapamycin.

### Correlation between clinicopathologic parameters and p-mTOR expression

The correlations between clinicopathological parameters and immunohistochemical expression of p-mTOR are summarized in Table 2. There was no correlation between the clinicopathological parameters and the p-mTOR expression.

**Table 2:** Associations between p-mTOR expression and clinicopathologic parameters in 160 patients with tongue squamous cell carcinoma.

Parameters		p-mTOR expression		
		Low	Over	P value
Age	≤52 y/o	42	40	0.26
	>52 y/o	33	45	
Pathological T classification	T1/2	44	54	0.53
	T3/4	31	31	
Pathological N classification	N0	40	47	0.80
	N1/2/3	35	38	
Pathological 7^th^ AJCC Stage	I/II	26	39	0.15
	III/IV	49	46	
Depth of invasion (mm)	<4 mm	8	12	0.51
	≥4 mm	67	73	
Histologic grade	1	42	51	0.61
	2/3	33	34	
Vascular invasion	Absent	66	66	0.085
	Present	9	19	
Perineural invasion	Absent	38	51	0.24
	Present	37	34	
Extracapsular spread	Absent	56	64	0.93
	Present	19	21	
Margin status	Negative	70	78	0.71
	Positive/close	5	7	
Smoking	Absent	14	15	0.87
	Present	61	70	
Alcohol	Absent	13	19	0.43
	Present	62	66	
Betel-nut chewing	Absent	20	19	0.53
	Present	55	66	

Abbreviation: p-mTOR, phosphorylated mammalian target of rapamycin. *Statistically significant. х^2^ test or Fisher’s exact test was used for statistically analyzed.

### Survival analyses

**Figure 2:**
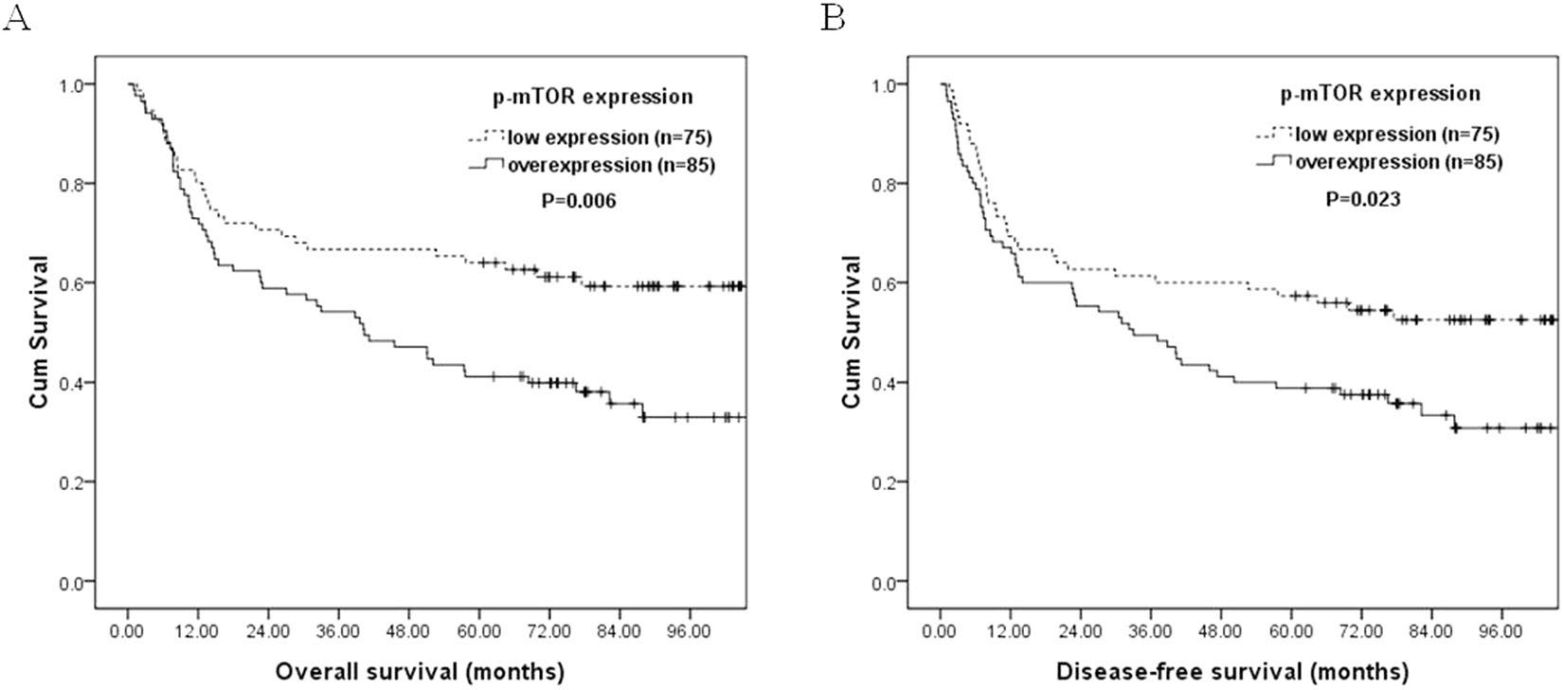
Kaplan–Meier plots to predict overall survival and disease-free survival according to p-mTOR protein immunoexpression. Tongue squamous cell carcinoma patients with p-mTOR overexpression have significantly shorter overall survival (**A**) and disease-free survival (**B**) than those with low p-mTOR expression.

Correlations of clinicopathological parameters and p-mTOR expression with overall survival and disease-free survival are shown in Table 3. Univariate analyses demonstrated that p-mTOR overexpression (P = 0.006, Fig. 2A), pathological T classification, T3/4 (P = 0.001), pathological N classification, N1/2/3 (P < 0.001), pathological 7^th^ AJCC Stage III/IV (P < 0.001), depth of invasion ≥4 mm (P = 0.046), vascular invasion (P = 0.007), perineural invasion (P = 0.025), extracapsular spread (P < 0.001), positive/close surgical margin (P = 0.003), and smoking history (P = 0.027) were significantly associated with inferior overall survival. Additionally, p-mTOR overexpression (P = 0.023, Fig. 2B), pathological T classification, T3/4 (P = 0.004), pathological N classification, N1/2/3 (P < 0.001), pathological 7^th^ AJCC Stage III/IV (P < 0.001), histologic grade 2/3 (P = 0.031), vascular invasion (P = 0.015), perineural invasion (P = 0.004), extracapsular spread (P < 0.001), positive/close surgical margin (P = 0.015), and smoking history (P = 0.042) were significantly associated with inferior disease-free survival.

**Table 3:** Results of univariate log-rank analysis of prognostic factors for overall survival and disease-free survival in 160 patients with tongue squamous cell carcinoma.

Factors	No. of patients	Overall survival (OS)		Disease-free survival (DFS)	
		5-year OS (%)	P value	5-year DFS (%)	P value
Age					
≤52 y/o	82	55%	0.31	52%	0.14
>52 y/o	78	49%		42%	
p-mTOR					
Low expression	75	64%	0.006*	57%	0.023*
Overexpression	85	41%		39%	
Pathological T classification					
T1/2	98	61%	0.001*	55%	0.004*
T3/4	62	37%		36%	
Pathological N classification					
N0	87	64%	<0.001*	61%	<0.001*
N1/2/3	73	37%		32%	
Pathological 7^th^ AJCC Stage					
I/II	65	68%	<0.001*	65%	<0.001*
III/IV	95	41%		36%	
Depth of invasion (mm)					
<4 mm	20	75%	0.046*	65%	0.11
≥4 mm	140	49%		45%	
Histologic grade					
1	93	57%	0.069	54%	0.031*
2/3	67	45%		39%	
Vascular invasion					
Absent	132	56%	0.007*	52%	0.015*
Present	28	32%		29%	
Perineural invasion					
Absent	89	58%	0.025*	56%	0.004*
Present	71	44%		37%	
Extracapsular spread					
Absent	120	59%	<0.001*	55%	<0.001*
Present	40	30%		25%	
Margin status					
Negative	148	54%	0.003*	49%	0.015*
Positive/close	12	25%		25%	
Smoking history					
Absent	29	72%	0.027*	66%	0.042*
Present	131	47%		44%	
Alcohol history					
Absent	32	56%	0.44	50%	0.52
Present	128	51%		47%	
Betel-nut chewing					
Absent	39	62%	0.1	54%	0.22
Present	121	49%		46%	

Abbreviation: p-mTOR, phosphorylated mammalian target of rapamycin; *Statistically significant.

In multivariate comparison, p-mTOR overexpression (P = 0.002, hazard ratio = 2.082, 95% confidence interval: 1.320–3.284) remained independently associated with worse overall survival, together with extracapsular spread (P = 0.012, hazard ratio = 1.920, 95% confidence interval: 1.157–3.187), and pathological 7^th^ AJCC Stage III/IV (P = 0.006, hazard ratio = 2.188, 95% confidence interval: 1.259–3.803). For disease-free survival, p-mTOR overexpression (P = 0.006, hazard ratio = 1.818, 95% confidence interval: 1.183–2.795), extracapsular spread (P = 0.011, hazard ratio = 1.876, 95% confidence interval: 1.152–3.054), pathological 7^th^ AJCC Stage III/IV (P = 0.004, hazard ratio = 2.195, 95% confidence interval: 1.294–3.726), and histologic grade 2/3 (P = 0.021, hazard ratio = 1.627, 95% confidence interval: 1.077–2.459) represented an independent adverse prognosticator. The 5-year overall and disease-free survival rates were 41% and 39% in patients with overexpression of p-mTOR, and 64% and 57% in patients with low expression of p-mTOR, respectively.

### Inhibition of endogenous mTOR by everolimus or mTOR siRNA reduces the viability of tongue cancer cells

**Figure 3:**
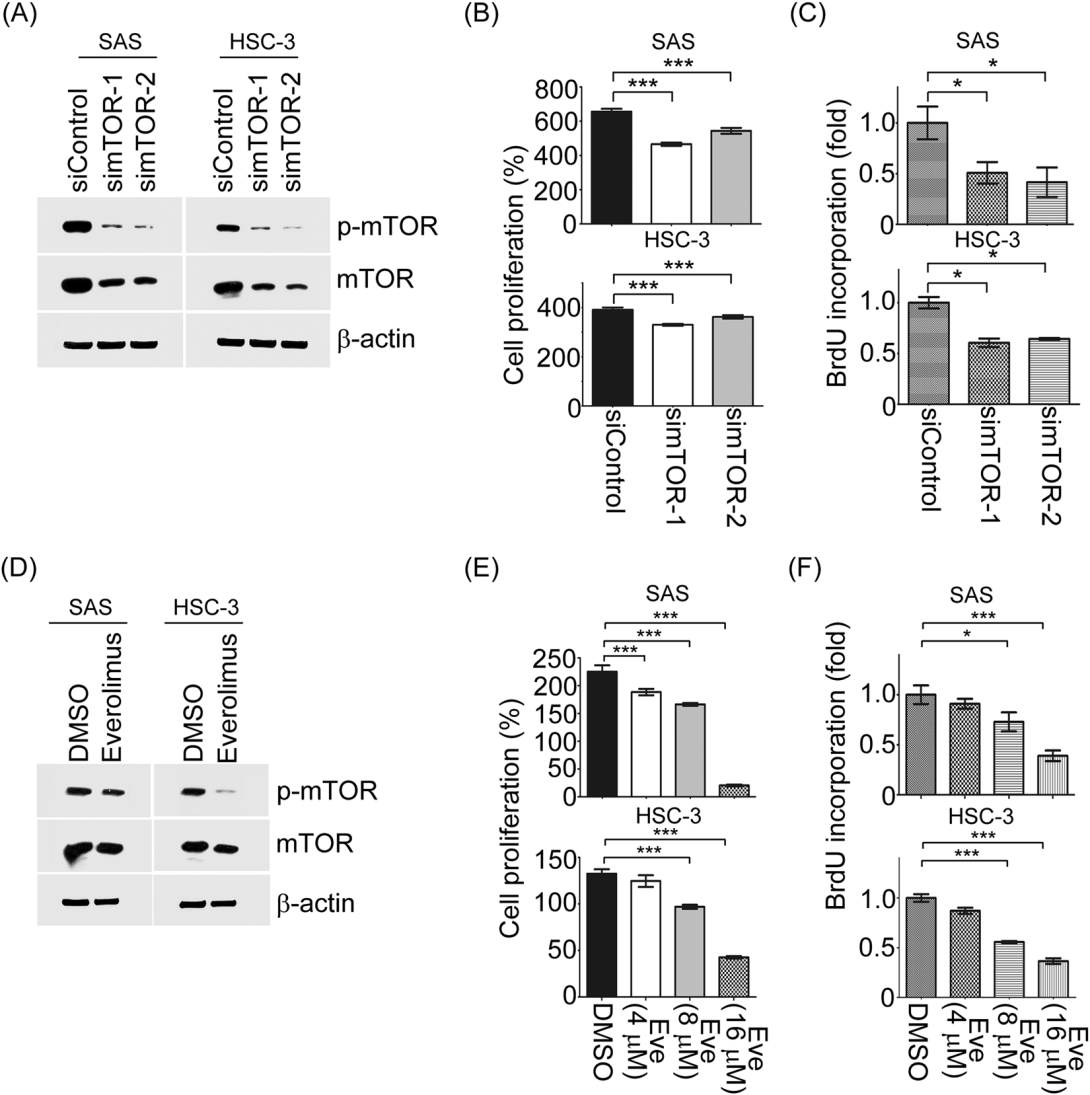
mTOR activity involved in cell proliferation of tongue cancer cells. (**A**) The protein expression levels of total mTOR, and phosphorylated mTOR were demonstrated in SAS and HSC-3 cells transfected with siControl and simTOR by Western blotting. (**B** and **C**) The cell growth abilities of siControl and simTOR in both SAS and HSC-3 cells were measured by MTT and BrdU assays. (**D**) The protein expression levels of total mTOR, and phosphorylated mTOR were detected in SAS and HSC-3 cells treated with everolimus or DMSO by Western blotting. (**E** and **F**) the cell growth abilities of both two cells treated with everolimus in a dose-dependent manner were assessed by MTT and BrdU assays. Eve: everolimus. *p < 0.05; **p < 0.01, ***p < 0.001.

First, we used siRNA to silence endogenous mTOR expression/or activity for functional studies in tongue cancer cells. Two specific mTOR siRNAs were transfected into SAS and HSC-3 cells; both effectively suppressed the expression of p-mTOR/mTOR (Fig. 3A). Using the same panel, we performed MTT and BrdU assays to determine the ability of cell growth. Both MTT and BrdU assays showed that simTOR transfectants suppressed the proliferation of SAS and HSC-3 cells significantly (Fig. 3B and C). To further confirm these results, cells treated with mTOR inhibitor, everolimus, was also performed. The mTOR activity was reduced in both tongue cancer cell lines upon everolimus stimulation by Western blotting (Fig. 3D). In agreement with result above, cell viability and BrdU incorporation were decreased while mTOR activity was inhibited (Fig. 3E and F). Taken together, these results demonstrated that mTOR expression/or activity plays a pivotal role in tumor growth of tongue cancer cells.

### Everolimus significantly decreased the incidence of tongue SCC in 4-NQO-induced tongue cancer murine model

**Figure 4:**
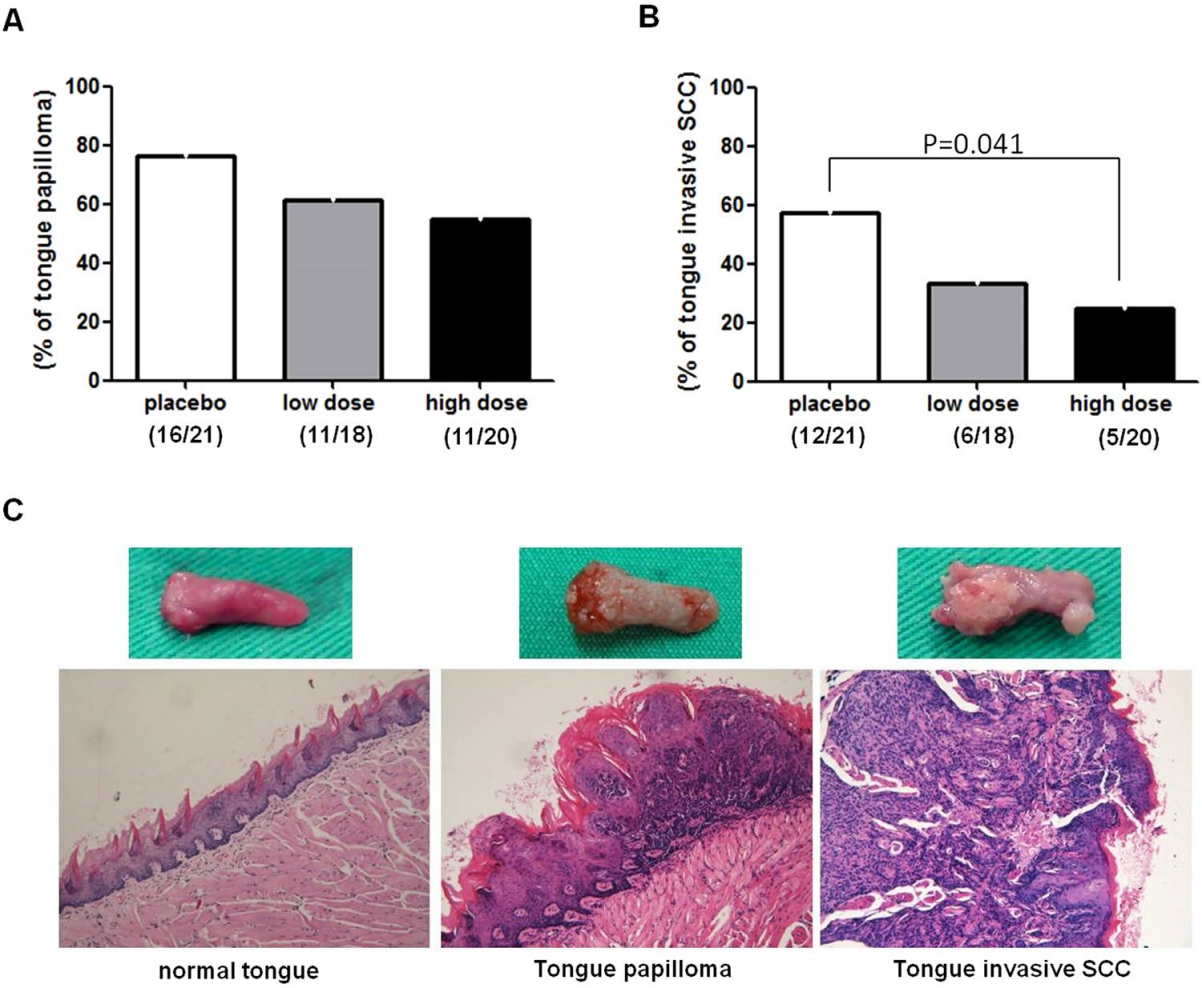
Inhibitory effect of the mTOR inhibitor, everolimus, in 4-NQO-induced tongue cancer murine model. (**A**) The incidence of tongue papilloma in mice treated with placebo, low dose everolimus, and high dose everolimus. There were no significant differences in papilloma between three groups. (**B**) The incidence of tongue SCC in mice treated with placebo, low dose everolimus, and high dose everolimus. Mice in high dose everolimus group developed significantly (P = 0.041) less invasive SCC than those in vehicle group. (**C**) Gross appearance and hematoxylin and eosin stained (**H** and **E**) sections of normal tongue, tongue papilloma, and tongue SCC from representative mice. Original magnification × 200. SCC: squamous cell carcinoma.

We next determined whether the inhibitory effect of mTOR inhibitor, everolimus, exists in 4-NQO-induced tongue cancer murine model. Among the 75 mice exposed to 4-NQO, 21 of the 25 mice in placebo group, 18 of the 25 mice in low-dose everolimus group, and 20 of the 25 mice in high dose everolimus group survived at the end of 28 weeks. Histopathologically, all of the surviving mice in three groups had hyperplasia and dysplasia. Papilloma was found in 16 of the 21 surviving mice in placebo group, 11 of the 18 surviving mice in low-dose everolimus group, and 11 of the 20 surviving mice in high-dose everolimus group. There were no significant differences in papilloma between three groups (Fig. 4A and C). Invasive SCC was found in 12 of the 21 surviving mice in placebo group, 6 of the 18 surviving mice in low-dose everolimus group, and 5 of the 20 surviving mice in high-dose everolimus group. Mice in high dose everolimus group developed significantly (P = 0.041, Fig. 4B and C) less invasive SCC than those in placebo group.

## Discussion

The activation of mTOR signaling pathway is common in solid tumors^7^. Numerous drugs have been developed to target different components of this pathway^10, 11^. However the prognostic value of mTOR signaling activation remained largely undefined in cancer patients. For head and neck cancer, Garcia -Carracedo *et al*.^12^ reported a positive correlation between the mTOR activation and a less aggressive phenotype, resulting in a significantly improved survival in patients with laryngeal cancer. Naruse *et al*.^13^ and Monteiro *et al*.^14^ showed that p-mTOR overexpression was correlated with poor survival rate in patients with oral SCC. These incongruous findings suggest that mTOR signaling may be various in different tumor site of head and neck cancer. Further studies on different tumor site of head and neck cancer are thus necessary to fully evaluate the effect of mTOR signaling activation. To the best of our knowledge, large series study focusing on tongue SCC only to assess the prognostic role of mTOR activation is lacking. Therefore, we conducted the present study. We found that p-mTOR overexpression was associated with inferior OS and DFS in 160 patients with tongue SCC, which further support previous findings from Naruse *et al*.^5^ and Monteiro *et al*.^14^ and highlight the important role of mTOR activation if tongue SCC.

Despite advance in imaging tool and surgical technique, there is still a proportion of patients with tongue SCC who develop recurrences after surgery. Thus, identification of patients at high risk for recurrence who may benefit from post-operative adjuvant therapy is worthwhile. In the present study, p-mTOR overexpression represented biological aggressive phenotype. The 5-year OS and DFS rates were 41% and 39%, respectively, in patients with p-mTOR overexpression, and 64% and 57%, respectively, in patients with low expression of p-mTOR, suggesting that p-mTOR status might be used to select some patients for adjuvant therapy after surgery. However, our clinical observation has ﻿an important limitation. It was a retrospective analysis.

Blockage of mTOR activity by an mTOR inhibitor, everolimus, exhibits an effect of anti-tumor development and is approved by the FDA for the treatment of several types of cancers^10^. The present study explored the effect of everolimus on tongue cancer cell lines. The results showed that everolimus significantly reduced the tumor growth and BrdU incorporation of SAS and HSC-3 cells. Furthermore, by using siRNA approach, the abilities of cell proliferation were attenuated in loss-of function of mTOR in both SAS and HSC. Altogether, these results suggest that silencing mTOR expression/activity may provide a potential strategy for the treatment of tongue squamous cell carcinoma.

In 4-NQO-induced tongue cancer murine model, we found that chronic administration of everolimus halts the malignant progression of carcinogen-induced tongue SCC. Raimondi *et al*.^15^ described that rapamycin prevents Early Onset of tongue SCC in an oral-Specific K-ras and p53 two-hit carcinogenesis mouse model. Pten ablation led to of PI3K/AKT/mTOR activation. Compared to control mice, Squarize *et al*.^16^ reported that intraoral administration of 4-NQO resulted in the faster development of oral-specific carcinomas in genetically defined mice displaying reduced PTEN expression achieved by the conditional deletion of PTEN using the keratin promoter 14 CRE-lox system. These studies from mice models further support the importance of the mTOR signaling pathway in the progression of tongue SCC.

Preclinical models of mouse with cancers are crucial tools for the study of testing novel therapeutic efficacy and treatment responses of drugs. The ideal model is cost effective to use, easily manipulable, and recapitulates the biology of human disease. To date, a seldom preclinical model of tongue cancer entirely executes all these criteria. 4-NQO, a synthetic water soluble carcinogen, has been used extensively to study oral carcinogenesis^17^. 4-NQO treatment of mice induced carcinoma in the tongue simulates many aspects of human tongue carcinogenesis, such as hyperplasia, dysplasia, severe dysplasia, *in situ* carcinoma and squamous cell carcinoma. 4-NQO-induced cancer model may have increased heterogeneity and genetic complexity of tumor cells, but it still has some limitations. For example, 4-NQO-induced tumor is not from human origin may not reflect relevant carcinogenic exposures and limit the utility as a preclinical platform for therapeutic research. In recent, patient-derived xenograft (PDX) mouse models are increasingly used for preclinical therapeutic testing of human cancer. PDX is based on transfer of human tumor tissue from a patient into immunosuppressed mice, with no interfering *ex vivo* cell culture^18^. PDX models retain the architecture and stromal components of the original tumor and preserve tumor heterogeneity. In addition, PDX model also provides the potential tool for the personalized drug therapy^19^. Despite the advantages of PDX, it still have some limitations, particularly, that an immunodeficient host is still required^20^. Our current study is limited that the therapeutic efficacy and response of everolimus by using PDX tongue cancer model should be further investigated in the future.

In conclusion, p-mTOR overexpression was independently associated with poor prognosis in patients with tongue SCC. Inhibition of mTOR activity showed the promising activity in tongue SCC cell lines and mouse model. Our results suggest that inhibition of mTOR signaling pathway may be a novel therapeutic target for the treatment of tongue SCC.

## Materials and Methods

### Patients and Samples

This study included 160 patients with available paraffin block who underwent primary surgical resection at Kaohsiung Chang Gung Memorial Hospital for treatment of tongue SCC. This retrospective study was approved by Chang Gung Medical Foundation Institutional Review Board. All procedures were conducted in accordance with guidelines provided by the Ethics Committees and Institutional Review Boards at Chang Gung Memorial Hospital. The informed consent was signed by all study participants. Patients with synchronous cancers in the other organ and patients receiving preoperative chemoradiotherapy, preoperative chemotherapy, or preoperative radiotherapy were excluded. Clinicopathologic information was obtained retrospectively from clinical records and pathology reports. The pathological TNM stage was determined according to the 7^th^ American Joint Committee on Cancer (AJCC) staging system^21^. Adjuvant therapy, such as radiation alone or concurrent chemoradiation with platinum-based chemotherapy, was used in patients with adverse pathologic features, which was mostly based on the National Comprehensive Cancer Network (NCCN) guidelines. The radiation technique was intensity-modulated radiation therapy. Overall survival (OS) was calculated from the time of surgery to death as a result of all causes. Disease-free survival (DFS) was computed from the time of surgery to the recurrence or death from any cause without evidence of recurrence.

### Immunohistochemistry

Immunohistochemistry staining was performed using an immunoperoxidase technique. Staining was performed on slides (4 mm) of formalin-fixed, paraffin-embedded tissue sections with primary antibodies against p-mTOR (Ser2448, Clone 49F9, 1:50, Cell Signaling Technology, Boston, MA, USA). Briefly, after deparaffinization and rehydration, the retrieval of the antigen was performed by treating the slides in 10 mmol/L citrate buffer (pH 6.0) in a hot water bath (95 °C) for 20 minutes. Endogenous peroxidase activity was blocked for 15 minutes in 0.3% hydrogen peroxide. After blocking with 1% goat serum for 1 hour at room temperature, the sections were incubated with primary antibodies for at least 18 hours at 4 °C overnight. Immunodetection was performed using the LSAB2 kit (Dako, Carpinteria, Calif) followed by 3–3′-diaminobenzidine for color development and hematoxylin for counterstaining. For p-mTOR, incubation without the primary antibody and normal esophageal squamous epithelium were used as a negative control, and normal gastric gland were used as a positive control^22, 23^.

The staining assessment was independently carried out by 2 pathologists (S.L.W. and W.T.H.) without any information about clinicopathologic features or prognosis. We followed the previously published method to score the expression of p-mTOR^8, 22, 23^. The fraction of tumor cells with significant staining (0–100%) was recorded, and the average value of 2 pathologists was calculated in each patient. The p-mTOR overexpression was defined as the presence of at least staining in ≧ 10% of tumor cells^8, 22^.

### Cell culture, MTT and BrdU assays

The tongue cancer cell lines SAS and HSC-3 were obtained from the ATCC and cultured in Dulbecco’s Modified Eagle Medium (DMEM) supplemented with 10% fetal calf serum, 2 mmol/L glutamine, 100 U/mL penicillin, and 100 µg/mL streptomycin. The viability of sub-confluent cells was analyzed by 3-(4,5-dimethylthiazole-2-yl)-2,5-diphenyltetrazolium bromide (MTT) reduction assay. For siRNA experiments, cell were seeded at 5 × 10^3^ cells/well in 96-well plates for culturing 48 h; for everolimus experiments, 5 × 10^3^ cells in 96-well plates treated with or without everolimus as indicated concentration for 48 h. After that the culture medium was removed and the cells were washed with PBS. Cells were then incubated with 0.5 mg/ml MTT, in culture medium without FBS, for 4 h at 37 °C in a 5% CO_2_ atmosphere. The medium was removed and 100 μL DMSO buffer was added and incubated in the dark for 10 min. Absorbance was measured on a microplate reader at 540 nm. The OD values were normalized with the value for the control group. For bromodeoxyuridine (BrdU) incorporation assay, cells were seeded at a density of 5 × 10^3^ cells/well in 96-well culture plates following treated with or without everolimus for 48 h. BrdU incorporation analysis was performed using the cell proliferation ELISA kit (Roche Diagnostics, Mannheim, Germany) according to the manufacturer’s instructions.

### 4-nitroquinoline 1-oxide (4-NQO)-induced Tongue Cancer Murine Model

The carcinogen, 4-NQO (Sigma Aldrich, St Louis, MO, USA) stock, was first dissolved in DMSO at 50 mg/mL as a stock solution and stored at −20 °C until used. On the days of 4-NQO administration, the stock solution was dissolved in propylene glycol (Sigma Aldrich, St Louis, MO, USA) and added to the drinking water bottles containing autoclaved tap water to obtain a final concentration of 100 μg/mL. All animal experiments were carried out in accordance with protocols approved by the Animal Use and Management Committee of Kaohsiung Chang-Gung Memorial Hospital. Fifty six-week-old C57Bl/6 mice were treated with 100 μg/ml 4-NQO in drinking water for 16 weeks and then resumed with normal autoclaved drinking water for another 12 weeks (total 28 weeks). Everolimus is the mTOR signaling pathway inhibitor. The treatment of the everolimus (low dose with 0.5 mg/kg/d for 5 days/wk or high dose with 5 mg/kg/d for 5 days/wk for 10 weeks by oral gavage)^24, 25^ or placebo control was started 16 weeks after initiation of 4-NOQ treatment, and each group had 25 mice. Mice dying prior to the end of the experiment were excluded from the analysis. At the end of the experiment, mice were sacrificed, and tongues were excised, fixed in 10% buffered formalin, embedded in paraffin blocks, and sectioned for hematoxylin and eosin staining. The histological determination were performed by two pathologists (S.L.W. and W.T.H.) according to the criteria described previously^17, 26^. The lesions observed were classified into four types: epithelial hyperplasia; dysplasia; papilloma; and invasive squamous cell carcinoma. Hyperplasia was defined as thickened epithelium with prominent surface keratinization and with or without elongated rete ridges. Dysplasia was defined as loss of polarity in the epithelial cells, nuclear pleomorphism and hyperchromasia, abnormal single cell keratinization (dyskeratosis), and increased or abnormal mitoses. Papilloma was defined as noninvasive exophytic growth of neoplastic cells, and invasive squamous cell carcinoma was defined as a lesion with invasion into the subepithelial tissues.

### Statistical Analysis

For patient data, statistical analysis was performed using the SPSS 17 software package. The Chi-square test or Fisher’s exact test were employed to compare data between the two groups. For survival analysis, the Kaplan–Meier method was used for univariate analysis, and the difference between survival curves was tested by a log-rank test. In a stepwise forward fashion, parameters with P value < 0.1 at univariate level were entered into Cox regression model to analyze their relative prognostic importance. For 4-NQO-induced tongue tumor mice experiments, statistical analyses of the incidence of tongue tumors were performed using *t* test. For all analyses, a P value < 0.05 was considered statistically significant.

### Ethics approval and consent to participate

This retrospective study was approved by Chang Gung Medical Foundation Institutional Review Board. All procedures were conducted﻿ in accordance with guidelines provided by the Ethics Committees and Institutional Review Boards at Chang Gung Me﻿morial Hospital. The written informed consent was obtained from patients.

### Data availability

The datasets supporting the conclusions of this article are included within the article.

## Electronic supplementary material

Supplementary information
