# Supplementary material for: Prognostic significance and function of mammalian target of rapamycin in tongue squamous cell carcinoma
Source: Sci Rep. 2017 Aug 15;7:8178. doi: 10.1038/s41598-017-08345-8 (PMC5558018; doi:10.1038/s41598-017-08345-8)
Supplement: Supplementary file 1 — Supplementary information [file 41598_2017_8345_MOESM1_ESM.pdf]

Title: Prognostic significance and function of mammalian target of rapamycin in tongue squamous cell carcinoma

Shau-Hsuan Li, MD[1], Chih-Yen Chien, MD, FACS[2], Wan-Ting Huang, MD[3], Sheng-Dean Luo, MD, FACS[2], Yan-Ye Su, MD[2], Wan-Yu Tien, Msc[1], Ya-Chun Lan, Msc[1], Chang-Han Chen, PhD[4,5,6]

[1]Department of Hematology-Oncology, Kaohsiung Chang Gung Memorial Hospital and Chang Gung University College of Medicine, Kaohsiung, Taiwan

[2]Department of Otolaryngology, Kaohsiung Chang Gung Memorial Hospital and Chang Gung University College of Medicine, Kaohsiung, Taiwan

[3]Department of Pathology, Kaohsiung Chang Gung Memorial Hospital and Chang Gung University College of Medicine, Kaohsiung, Taiwan

[4]Institute for Translational Research in Biomedicine, Kaohsiung Chang Gung Memorial Hospital, Kaohsiung, Taiwan

[5]Department of Applied Chemistry, and Graduate Institute of Biomedicine and Biomedical Technology, National Chi Nan University, Taiwan

[6]Center for Infectious Disease and Cancer Research, Kaohsiung Medical University, Kaohsiung, Taiwan

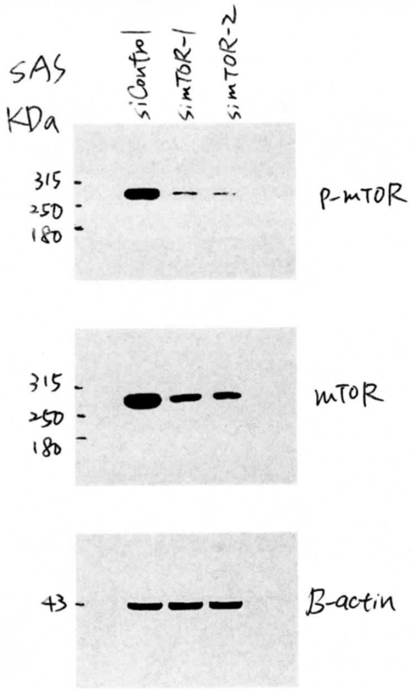
SAS
KDa
siControl
simTOR-1
simTOR-2
315
250
180
P-mTOR
315
250
180
mTOR
43
β-actin

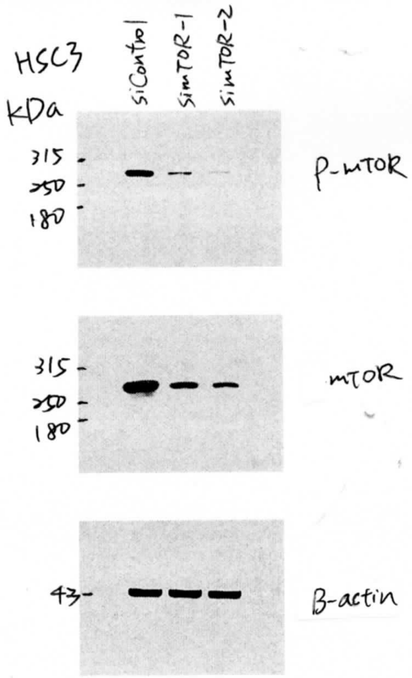
HSC3
KDa
siControl
simTOR-1
simTOR-2
315
250
180
P-mTOR
315
250
180
mTOR
43
β-actin

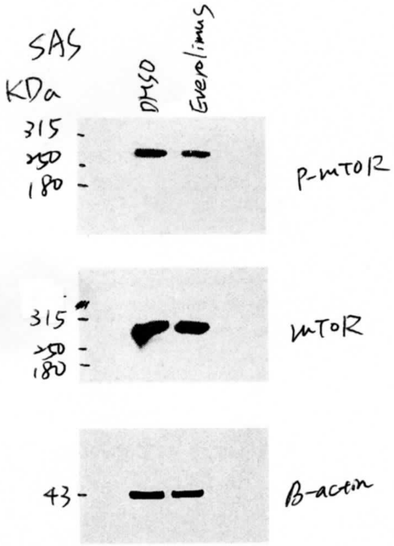
SAS
KDa
DMSO
Everolimus
315
250
180
P-mTOR
315
250
180
mTOR
43
β-actin

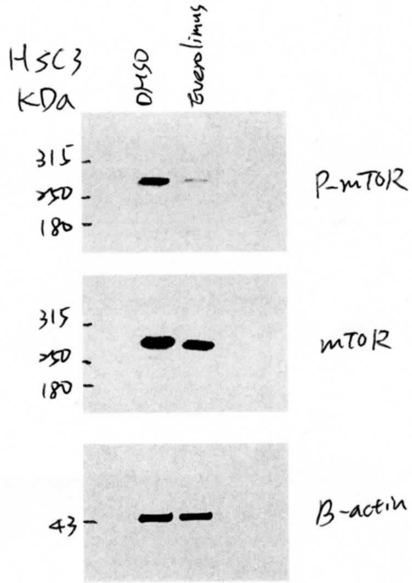
HSC3
KDa
DMSO
Everolimus
315
250
180
P-mTOR
315
250
180
mTOR
43
β-actin
